# RNA-Seq time-course analysis of neural precursor cell transcriptome in response to herpes simplex Virus-1 infection

**DOI:** 10.1007/s13365-024-01198-8

**Published:** 2024-03-13

**Authors:** Joel A. Wood, Srilakshmi Chaparala, Cecilia Bantang, Ansuman Chattopadhyay, Maribeth A. Wesesky, Paul R. Kinchington, Vishwajit L. Nimgaonkar, David C. Bloom, Leonardo D’Aiuto

**Affiliations:** 1 Western Psychiatric Institute and Clinic, Department of Psychiatry, University of Pittsburgh School of Medicine, 3811 O’Hara Street, 15213 Pittsburgh, PA USA; 2 Molecular Biology Information Service, Health Sciences Library System / Falk Library, University of Pittsburgh, M722 Alan Magee Scaife Hall / 3550 Terrace Street, 15261 Pittsburgh, PA USA; 3 Department of Ophthalmology, University of Pittsburgh, Suite 820, Eye & Ear Building, 203 Lothrop Street, 15213 Pittsburgh, PA USA; 4 VA Pittsburgh Healthcare system at U.S. Department of Veterans Affairs, Pittsburgh, PA USA; 5 Academic Research Building, Department of Molecular Genetics and Microbiology, University of Florida, 1200 Newell Drive, R2-231, 32610 Gainesville, FL USA

**Keywords:** Neurospheres, Herpes simplex virus (HSV), Human induced pluripotent stem cells, (hiPSCs), Neural precursor cells, RNA-Seq, Time-course analysis

## Abstract

The neurogenic niches within the central nervous system serve as essential reservoirs for neural precursor cells (NPCs), playing a crucial role in neurogenesis. However, these NPCs are particularly vulnerable to infection by the herpes simplex virus 1 (HSV-1). In the present study, we investigated the changes in the transcriptome of NPCs in response to HSV-1 infection using bulk RNA-Seq, compared to those of uninfected samples, at different time points post infection and in the presence or absence of antivirals. The results showed that NPCs upon HSV-1 infection undergo a significant dysregulation of genes playing a crucial role in aspects of neurogenesis, including genes affecting NPC proliferation, migration, and differentiation. Our analysis revealed that the CREB signaling, which plays a crucial role in the regulation of neurogenesis and memory consolidation, was the most consistantly downregulated pathway, even in the presence of antivirals. Additionally, cholesterol biosynthesis was significantly downregulated in HSV-1-infected NPCs. The findings from this study, for the first time, offer insights into the intricate molecular mechanisms that underlie the neurogenesis impairment associated with HSV-1 infection.

## Introduction

In 1995, Becker made a prescient proposition, indicating that latent infection with herpes simplex virus 1 (HSV-1) might elicit deleterious and unanticipated consequences on human cognition and behavior (Becker 1995; Ando et al. 2008). This hypothesis gains plausibility in light of evidence that the virus induces damage in brain regions associated with memory formation, including the hippocampus and associated limbic structures (Beers et al. 1995). An increasing body of literature has shed light on the potential mechanisms involved in the impact of HSV-1 on cognition and behavior.

Adult neurogenesis is the process of generating new neurons in the adult brain, which occurs throughout the lifespan of an individual (Ming and Song 2011). This process occurs in neurogenic niches of the brain, including the subgranular zone (SGZ) of the hippocampus and the subventricular zone (SVZ) lining the walls of the lateral ventricles, which are enriched with neural precursor cells (NPCs) (Jurkowski et al. 2020; Niklison Chirou et al. 2020; Dillen et al. 2020). A neurogenic niche represents residual segment of the embryonic germinal layer region possessing a unique and specialized microenvironment that sustain NPCs, and exerts a precise control over their activity, leading to the occurrence of adult neurogenesis (Kriegstein and Alvarez-Buylla 2009). The new neurons generated from NPCs residing in these regions contribute to learning and memory (Park et al. 2015). Besides SVZ and SGZ, recent studies have suggested the presence of additional neurogenic areas in other brain regions, including the hypothalamus, striatum, substantia nigra, cortex, and amygdala (Jurkowski et al. 2020).

HSV-1 exhibits a preference toward SVZ and SGZ (Yong et al. 2021; Menendez et al. 2016) and it can impact neurogenesis by impairing NPC proliferation, self-renewal, and migration(Zheng et al. 2022; Li Puma et al. 2019), all leading to impaired neuronal differentiation (LiPuma et al. 2019; Qiao et al. 2020). NPCs support productive HSV-1 infection (Zheng et al. 2020b; LiPuma et al. 2019). Dysregulation of the molecular mechanisms governing neurogenesis can give rise to significant consequences on cognition, encompassing cognitive decline (Toda et al. 2019), behavioral phenotypes (Tunc-Ozcan et al. 2019), and interference with hippocampus-dependent processing and behavior (Li Puma et al. 2020). We have previously shown that HSV-1 induces alterations in cognition-related pathways, such as glutamate and cAMP response element-binding protein (CREB) signaling (D’Aiuto et al. 2014).

Even though the advent of acyclovir therapy has significantly reduced the rate of mortality to approximately 25% in patients with HSV-1 encephalitis (HSE), patients who survive often experience significant long-term sequelae. Among acyclovir-treated HSE survivors, over 60% experience severe neurologic deficits. Memory, both anterograde and retrograde, is often impaired even with successful treatment of HSE (Bradshaw and Venkatesan 2016). Executive function and language ability also may be impaired (Jonker et al. 2014). The chronic lesions in HSE patients are mainly in the limbic system, which includes the hippocampus. The severity of these sequelae is related to the severity of damage to these limbic structures and on the patient’s age and neurologic status at the time of diagnosis. Despite the administration of antiviral treatment, the underlying cause for the persistence of these sequela remains poorly understood.

In the present study, we aimed to gain insights into how HSV-1 might disrupt neurogenesis at the molecular level and investigate the efficacy of antivirals to prevent the dysregulation of pathways playing important roles in neurogenesis. To achieve this, we conducted a time-course RNA-seq analysis on uninfected and infected neurospheres at three time points, both in the presence and absence of antivirals, employing two different multiplicities of infection (MOIs), with three replicates in each condition. The rational of this dual-MOI approach is that if specific pathways are dysregulated consistently in the same direction across both MOIs, it would provide evidence that these changes are biologically meaningful. The choice to employ antiviral (E)-5-(2-bromovinyl)-2’-deoxyuridine (5BVdU) and interferon-α (IFN-α) stems from our previously reported observation that acyclovir shows reduced antiviral efficacy in HSV-1 infected NPCs as compared to 5BVdU + IFN-α (Zheng et al. 2020b). The choice to utilize bulk RNA-Seq instead of single cell RNAseq was based on the fact that the former offers higher coverage of the transcriptome compared to the latter. Additionally, bulk RNA-seq provides a comprehensive overview of gene expression changes within a sample, making it a valuable tool for identifying pathways that are affected by HSV-1 infection and antiviral treatment (Haque et al. 2017).

Our analysis has revealed a distinct set of genes regulating the neurogenesis that are dysregulated during HSV-1, as well as genes whose expression remains altered even in the presence of antivirals. In addition, we provide evidence of downregulation in infected NPCs of several genes in the cholesterol biosynthesis network, which has been hypothesized representing a host antiviral defense mechanism (Blanc et al. 2011; Wang et al. 2020; Sviridov and Bukrinsky 2014).

## Results

### RNA-Seq time-course analysis of NPCs transcriptome in response to HSV-1 infection

**Fig. 1:**
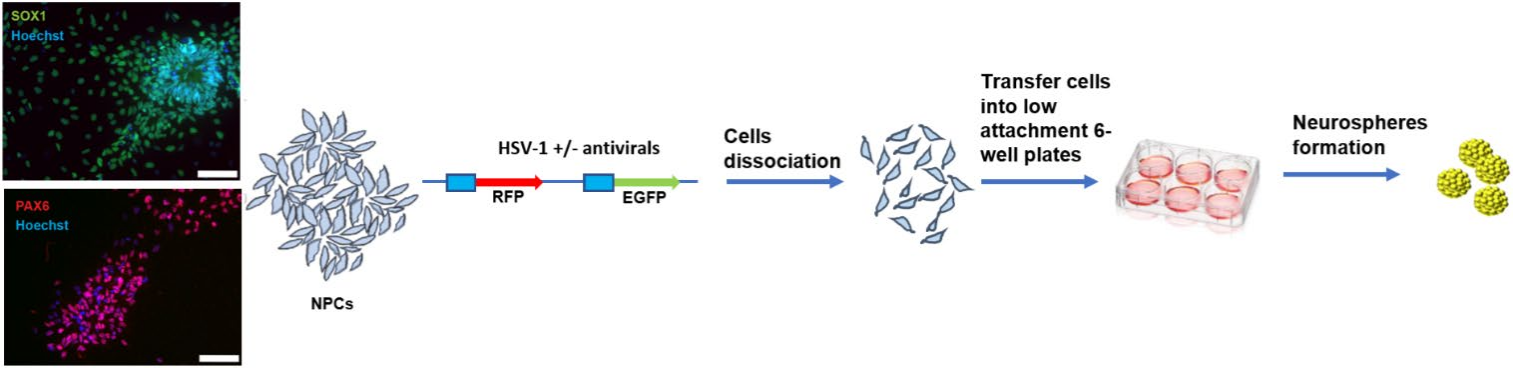
NPCs were infected using a dual florescent reporter HSV-1 at MOI 0.001 ad 0.0001, with and without the presence of antivirals 5BVdU + IFN-alpha. Cells were then dissociated and transferred into a 6-well low attachment plate where neurospheres were grown, then analyzed at day 3, 5, and 7

Our previous work described the impairment in proliferation, migration, and differentiation of NPCs infected with HSV-1 using a 3D model of NPCs (neurospheres) (Zheng et al. 2020a, 2022). To investigate the changes in transcription in human NPCs following HSV-1 infection, we conducted a time-course RNA-Seq study using NPCs-derived neurospheres. The method for generating NPCs from human iPSCs is detailed in the methods section. NPCs were infected with a genetically engineered HSV-1 KOS strain that expresses enhanced green fluorescent protein (EGFP) and red fluorescent protein (RFP) under the control of immediate early and late gene promoters, respectively (Zheng et al. 2020a). Cells were infected at the multiplicity of infections (MOIs) of 0.001 and 0.0001 in the presence or absence of antivirals 5BVdU + IFN-α. After one hour, the inocula were removed, cells were washed and dissociated, and were transferred into low attachment 6-well plate for the generation of neurospheres. Uninfected and infected neurospheres were harvested at days 3, 5, and 7 post-infection (p.i.) (Fig. 1). The choice of the MOIs relies on the evidence that antivirals 5BVdU + IFN-α (which demonstrate greater efficacy when compared to acyclovir) cannot effectively inhibit viral replication at MOIs higher than 0.001 across 7 days following infection (Zheng et al. 2020a). The expression of the fluorescent reporter genes at the different time points confirmed the infection of NPCs (data not shown).

**Fig. 2:**
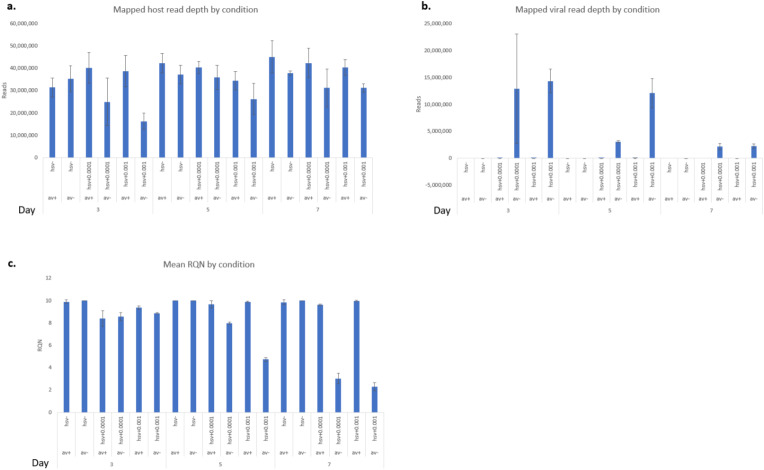
Panel **a**: Mean number of Host RNA-seq reads mapped for triplicate samples in each condition, following QC. Panel **b**: Mean number of viral RNA-seq reads mapped for samples in each condition. Panel **c**: Average RNA RQN for samples in each condition. Data presented for each condition represents the mean of the replicates, with error bars representing the standard deviation. Av + = with antivirals, Av-= without antivirals, hsv + = infected, hsv-=uninfected, 0.001 and 0.0001 are the MOIs in infected cells

**Fig. 3:**
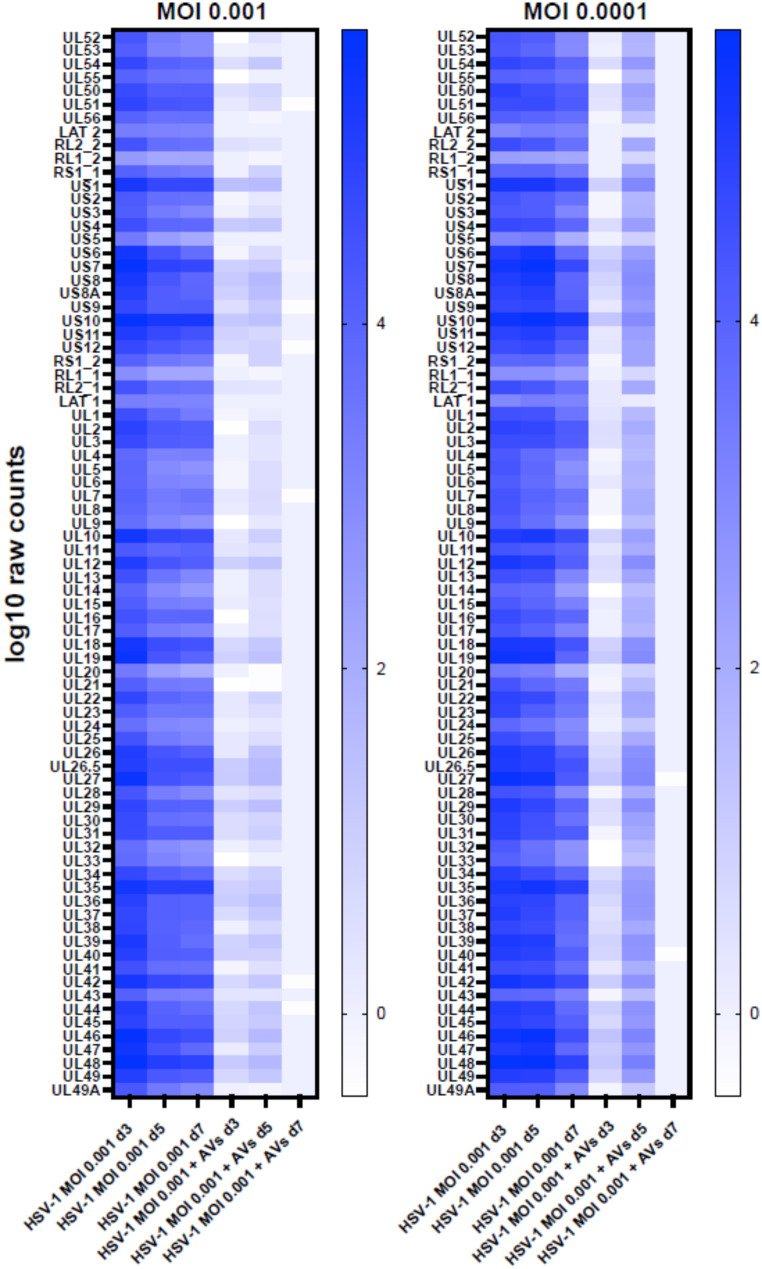
Expression of viral genes in NPCs infected at MOIs of 0.001 and 0.0001. The color bar represents log10 of transcript counts. Av + = with antivirals, Av-= without antivirals

RNA-Seq mapping results revealed a range of nucleotide coverage, from 24.6 million reads (day 7, MOI 0.0001 condition) to 51.2 million reads (day 7, uninfected condition in the presence of antivirals), as shown in Fig. 2a. Mapped reads ranged from 12.6 million reads to 50.2 million reads (mean 34.9, sd 8.4). Following human transcript mapping, the remaining unmapped reads were collected and mapped to the Human Herpesvirus strain KOS genome (GenBank: JQ780693.1) in a similar manner. Our analysis revealed a strong impact of antivirals (Avs) 5BVdU + IFN-α on HSV-1 infection during days 3 to 7 post-infection, with the most significant results observed on day 7, where infected NPCs exposed to the antiviral had a minimal number of viral transcripts (Figs. 2b and 3). An increase in the viral genes was detected on day 5 post-infection, suggesting a temporary surge in virus production. A total of 12,484 human genes were expressed at mean transcripts per million (TPM) > = 5 in uninfected neurospheres untreated with Avs in at least 1 condition, and 12,610 in uninfected neurospheres treated with Avs in at least 1 condition. Details of the RNA-Seq experiments can be found in the methods section.

**Fig. 4:**
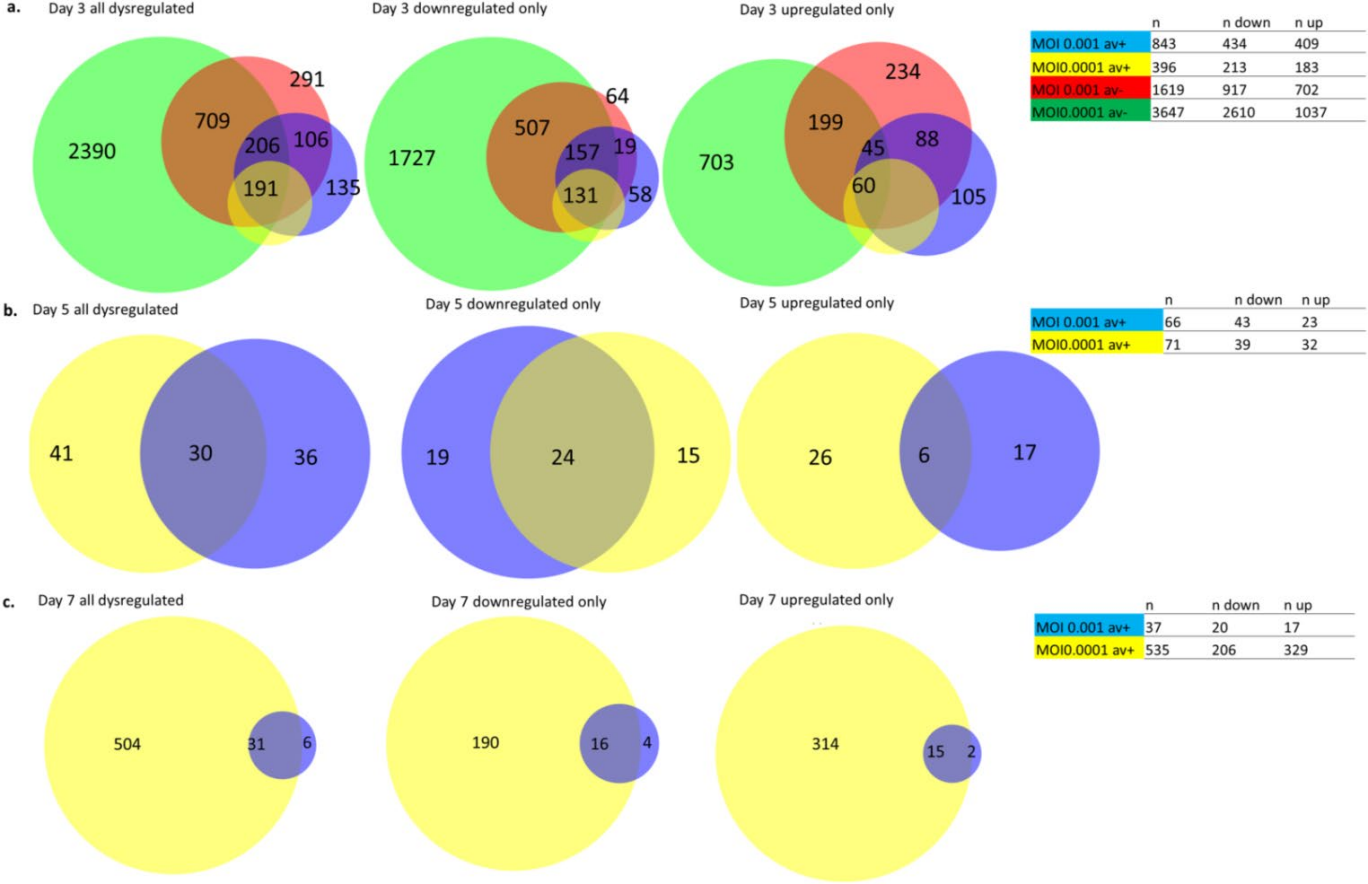
Panel **A**: Overlap of genes dysregulated across all conditions at day 3, 5, and separately those that are downregulated and those that are upregulated. Panel **B**: Overlap of Genes dysregulated across all conditions at day 5 and separate those that are downregulated and those that are upregulated. Panel **C**: Overlap of genes dysregulated across all conditions at day 7 and separate those that are downregulated and those that are upregulated. For all, dysregulation is defined as FDR *p* < 0.5 (edgeR test) and expression TPM ≥ 5. Blue = MOI 0.001 Av+, yellow = MOI 0.0001 Av+, red = MOI 0.001 Av-, green = MOI 0.0001 Av-, Av=antiviral

In infected neurospheres, the number of differentially expressed host genes ranged from 37 − 3,647 at the three time points in the different conditions when compared to uninfected cells, defined as FDR corrected significance at p < = 0.05 based on edgeR analysis and max group mean of > = 5 TPM in expression (Fig. 4). At one or more of the three time points, a total of 4,547 genes demonstrated differential expression. Comparisons within days 5 and 7 in the absence of Avs were not considered reliable because several conditions on those days exhibited unacceptable RNA degradation based on QC (Fig. 2c).

The differentially expressed genes (DEGs) were categorized into up-regulated and down-regulated groups, with 2,652 unique genes being exclusively down-regulated across conditions, 1,601 genes being exclusively up-regulated, and 294 exhibiting a mixed profile (Fig. 4). Several genes were only differentially expressed at one of the three sampling times. Out of these, a greater number of genes (933) were differentially expressed at 3 dpi compared to 5 or 7 dpi in the presence of antivirals.

**Fig. 5:**
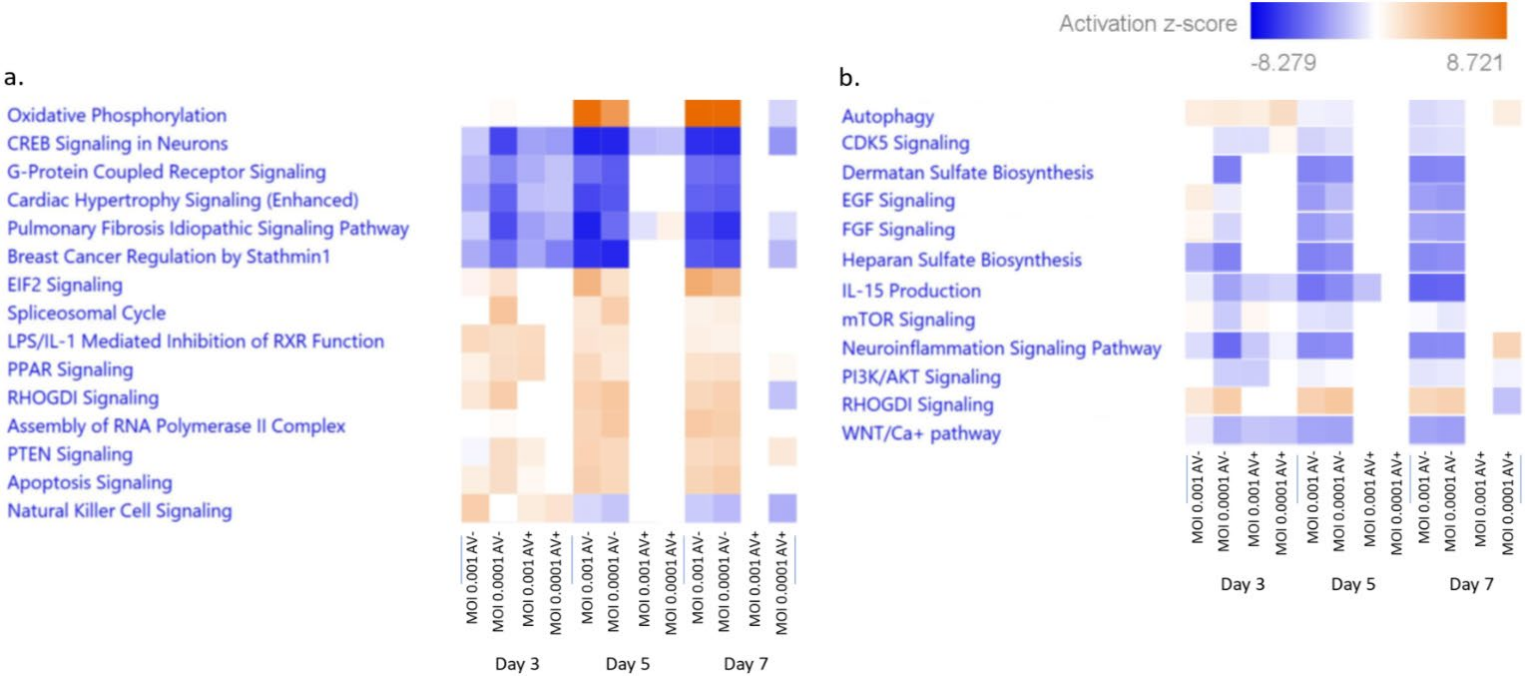
Differentially expressed genes (DEG) measured by comparing infected cells vs. uninfected cells and infected cells treated with antivirals vs. uninfected cells cultured in the presence of antivirals were subjected to Ingenuity Pathway Analysis (IPA). Activation (+ z score, orange boxes) or inhibition (-z score, blue boxes) of each pathway is a measure of experimentally determined gene expression changes reported in literature. The intensity of the color indicates the degree of activation/ inhibition. Panel **a**: Top fifteen hierarchically clustered significantly dysregulated pathways. Panel **b**: Important selected pathways significantly dysregulated by HSV-1 infection of NPCs. Av-: Without antivirals; Av+: With antivirals

We employed Ingenuity Pathway Analysis (IPA) to determine the top ten hierarchically clustered, significantly dysregulated canonical pathways that were significantly altered in response to HSV-1 infection in the presence or absence of antivirals (Fig. 5). Statistical significance was calculated using right-tailed Fisher Exact Probability Tests; biological pathways showing p-value < 0.05 were considered statistically significant. The fifteen most significantly altered pathways in neurospheres infected in the presence or absence of antiviral are shown in Fig. 5a. According to IPA’s canonical pathway analysis of these commonly DEGs, oxidative phosphorylation, EIF2, apoptosis, assembly of RNA polymerase, and RHOGDI were generally found to be upregulated (Fig. 5). Conversely, CREB signaling, IL-15 production, WNT/Ca + pathway, neuroinflammation signaling, heparan sulfate, dermatan sulfate, and cholesterol biosynthesis were generally downregulated (Fig. 5).

The apoptosis signaling pathways exhibited upregulation across all time points at both multiplicities of infection (Fig. 5a). Autophagy exhibited upregulation at day 3 post-infection. However, a downregulation of autophagy was observed at days 5 and 7 post-infection (Fig. 5b). The PI3K/AKT signaling cascade exerts a suppressive effect on the lytic cycle of viral replication by maintaining a repressive chromatin state of the viral genome via the activation of eIF4E-binding protein (4E-BP). A key component of this pathway is the mammalian target of rapamycin (mTOR) (Kobayashi et al. 2012). Notably, in acutely infected neurospheres, it was observed that both the PI3K/AKT pathway and mTOR are downregulated (Fig. 5b).

### Effect of HSV-1 on NPC functions

Based on IPA analysis, we next aimed to identify the genes and/or pathways that impair neuroectodermal formation and the different aspects of NPC neurogenesis. The inhibition of TGF-b and BMP signaling pathways have been shown to induce neuroectodermal formation. In the context of HSV-1 infection of neurospheres, it has been observed that these signaling pathways are downregulated. Consequently, it could indicate that HSV-1 infection may, in principle, influence neuroectoderm formation. However, it is important to note that the inhibition of BMP signaling has been reported to initiate neural induction through the activation of fibroblast growth factor (FGF) signaling and *ZIC* genes (Marchal et al. 2009). Remarkably, our results show a downregulation of the FGF signaling in neurospheres infected at both MOIs. These findings suggest that HSV-1 could potentially influence neuroectodermal formation.

**Fig. 6:**
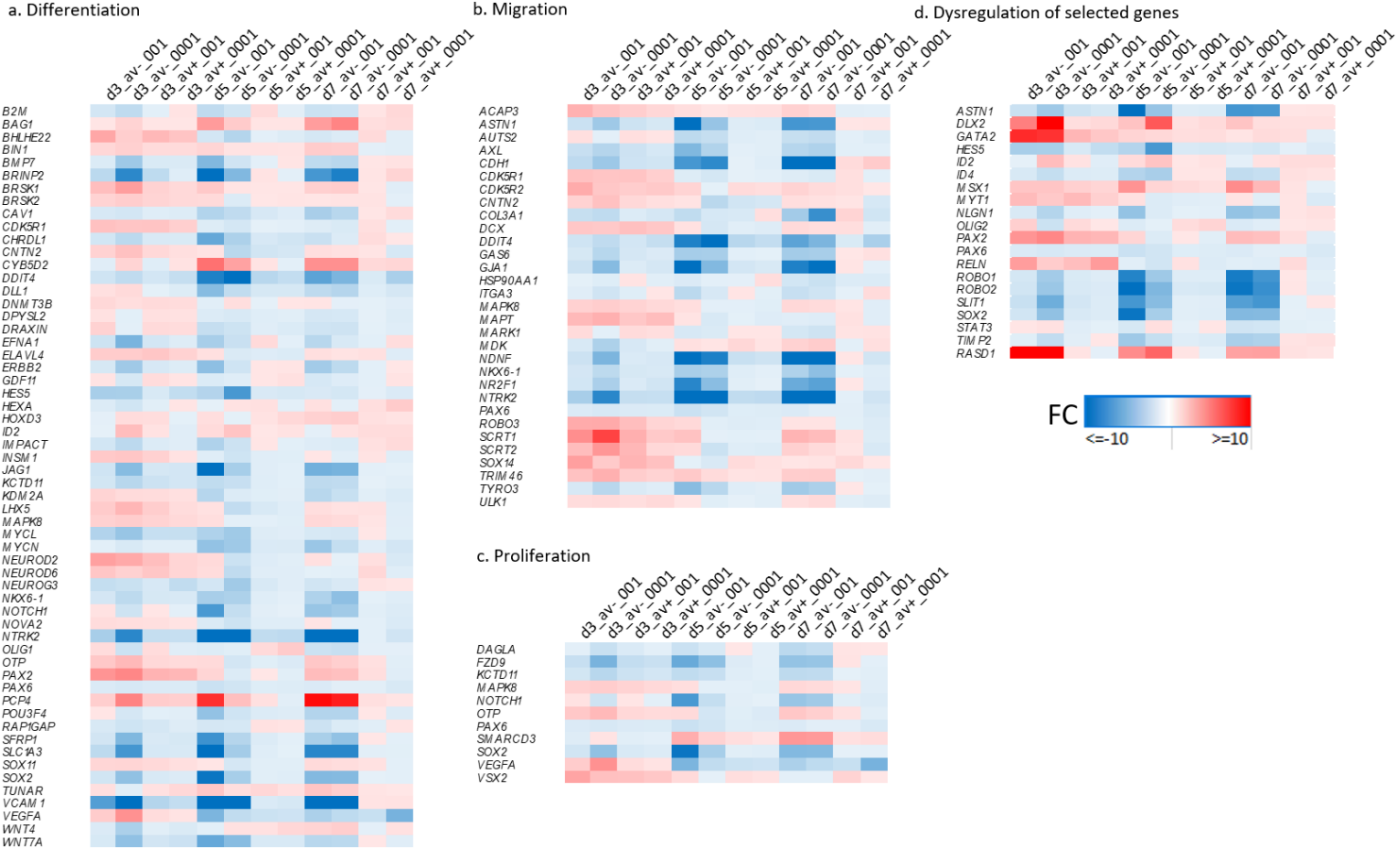
Specific genes dysregulated at MOI 0.001 and 0.0001, with and without antiviral 5BVdU + IFNα-, over day 3, 5, and 7. Panel **a**: The most significantly dysregulated genes involved in NPC differentiation. Panel **b**: The most significantly dysregulated genes involved in NPC migration. Panel **c**: The most significantly dysregulated genes involved in NPC proliferation. Panel d: Dysregulation of selected genes involved in maintenance, self- renewal, differentiation, and migration. D3, d5, d7 = day 3, 5 and 7; Av + = with antivirals, Av-=without antivirals, 001 = MOI 0.001, 0001 = MOI 0.0001; FC = fold change

**Fig. 7:**
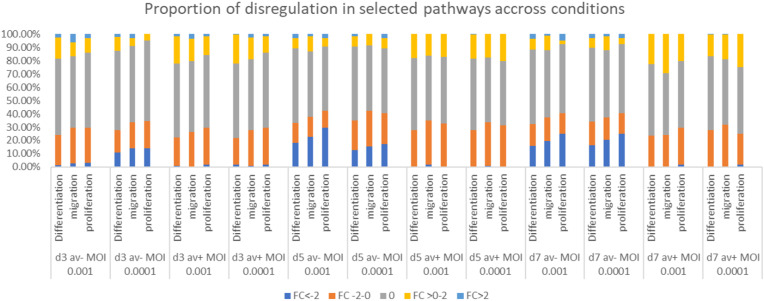
Proportion of genes in each condition exhibiting dysregulation (where TPM > = 5) in bins of: FC <-2, FC between − 2 and 0, FC between 0 and 2, and FC > 2. Genes expressing with a mean < TPM 5 in the relevant conditions are set at 0

Our previous findings have shown the negative impact of HSV-1 on proliferation, self-renewal, and migration abilities of NPCs (Zheng et al. 2022). To gain insights into the underlying mechanisms, we analyzed the RNA sequencing data of infected neurospheres at the different time points and MOIs to identify potential candidate genes responsible for these impairments. Our analysis indicated that a number of crucial pathways are downregulated in infected cells. These include: the *WNT*/β-catenin pathway, which is known to be active in the ventricular zone during cortical development and plays a crucial role in regulating the proliferation of NPCs (Kuwahara et al. 2014) (Fig. 5b); the Notch signaling pathway, which is essential for the maintenance of neural stem cells (NSCs) by enhancing the NSC self-renewal and by inhibiting differentiation (Wang et al. 2009; Hitoshi et al. 2002; Lasky and Wu 2005); ephrin-B signaling, which seems to be important for regulating self-renewal (Qiu et al. 2008); the sonic hedgehog signaling pathway, which coordinates the proliferation and differentiation of neural stem/progenitor cells through its regulation of the cell cycle kinetics of radial glial cells and intermediate progenitor cells (Komada 2012). In addition, we compiled gene ontology (GO) gene sets related to NPC proliferation, migration and differentiation, and assessed dysregulation patterns in these genes. We observed that antivirals did not normalize upregulation of several genes involved in these processes. This suggests that the antiviral treatment had a more targeted or specific impact on the regulation of these groups of genes (Figs. 6 and 7).

A number of key genes involved in aspects of neurogenesis and maintenance were also downregulated: *NR2F1* plays a role in controlling the long-term self-renewal of neural progenitor cells by regulating cell cycle genes and key master genes involved in cortical development, such as *PAX6* (Fig. 6a-d) (Bertacchi et al. 2020); *STAT3*, which regulates the maintenance (Yoshimatsu et al. 2006), proliferation and differentiation of NPCs (Su et al. 2020); the high-mobility group DNA binding gene *SOX2* (Fig. 6a and c-d), which is an important factor for the maintenance of neural stem cells in adult neurogenic areas, and its regulatory mutations cause neurodegeneration and affects adult neurogenesis (Ferri et al. 2004); epidermal growth factor (*EGF*) and fibroblast growth factor (*FGF*) signaling pathway genes, which are crucial in regulating the proliferation of neural stem cells (Fig. 5b) (Vescovi et al. 1993; Gritti et al. 1996); *HES5*, involved in the maintenance of neural stem cells (Fig. 6a and d) (Ohtsuka et al. 2001).

Genes regulating the migration of early-born neurons, such as *ROBO1* (Gonda et al. 2013) *ROBO2* (Guerrero-Cazares et al. 2017), *SLIT1* (Deboux et al. 2020), and *ASTN1* (Wilson et al. 2010) were downregulated (Fig. 6d). Slit-Robo signaling has also been found to play a role in the regulation of the NPCs proliferation as well as cell branching and elongation (Borrell et al. 2012; Andrews et al. 2008). Conversely, Reelin, which regulates NPCs migration (Courtès et al. 2011), was found upregulated by HSV-1 infection. *GATA2*, which activates genes specific to the GABAergic neuron subtype in the midbrain (Lahti et al. 2013), and *PAX2*, which is involved in the specification of interneurons in the dorsal horn (Batista and Lewis 2008), are upregulated at both MOIs, but are both downregulated by day 7 (Fig. 6d). *RASD1*, proposed to exert an antiviral activity (Wyler et al. 2019) was found significantly upregulated on day 3 p.i. at both MOIs.

**Fig. 8:**
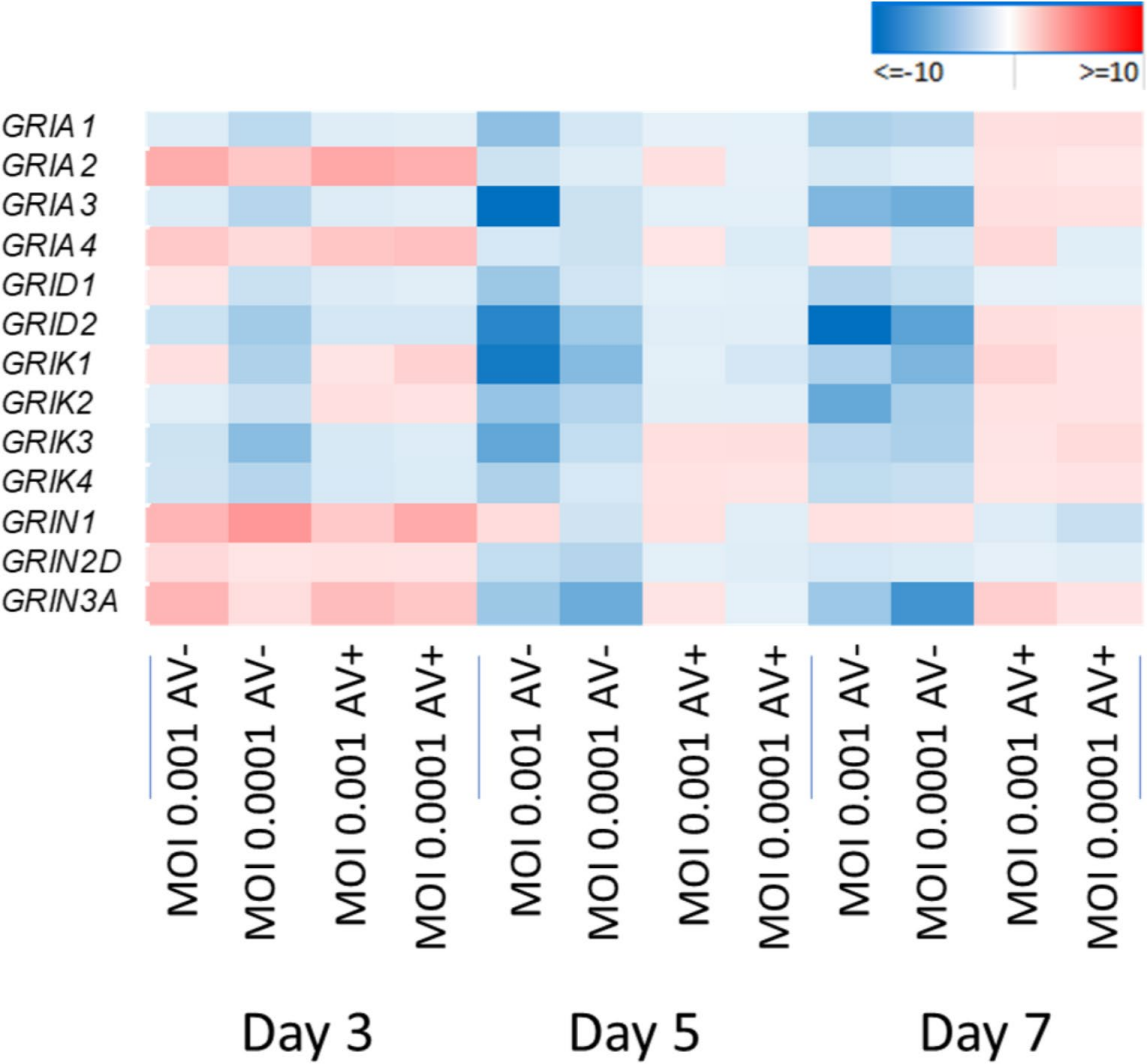
HSV-1 effects on glutamate inotropic receptors AMPA type, Delta, Kainate, and NMDA. The color scale represents FC in each condition

*MSX1*, whose overexpression in mouse neurospheres promotes oligodendrogenesis (Roybon et al. 2008), is overexpressed at day 3 post-infection (p.i.) at both MOIs (Fig. 6d). *HES5*, which inhibits both astrocyte and oligodendrocyte differentiation (Wu et al. 2003), is downregulated at both MOIs but upregulated at day 7 p.i. (Figure 6a and d). *MYT1*, which is involved in proliferation and differentiation of oligodendrocytes precursor cells (OPCs) (Wu et al. 2003), is upregulated at day 3 (Fig. 6d). *OLIG2*, which regulates key aspects of the oligodendrocytes development (Zhang et al. 2022), is upregulated at MOI 0.001 and downregulated at MOI 0.0001 (Fig. 6d). *ID4*, which inhibits OPC differentiation, is downregulated at both MOIs (Fig. 6d). Taken together, these results indicate that HSV-1 infection may favor oligodendrocyte differentiation from NPCs. However, *ID2*, which inhibits OPC differentiation (Emery and Lu 2015), is upregulated at day 3 at MOI 0.0001. Furthermore, *DLX2*, a modulator of neurons versus oligodendrocytes development in the ventral embryonic forebrain (Petryniak et al. 2007), is upregulated at day 3 at both MOIs. Overall, the net effect of the opposing gene expressions on oligodendrocyte differentiation of HSV-1-infected NPCs is uncertain. In HSV-1-infected NPCs, various genes that regulate neuronal differentiation and maturation were found to be dysregulated (Fig. 6). These include *NLGN1*, which induces neurite outgrowth (Fig. 6d) (Gjørlund et al. 2012), *PAX6* (Fig. 6a-d) (Sansom et al. 2009), and *TIMP2* (Fig. 6d) (Pérez-Martínez and Jaworski 2005), which promotes neuronal differentiation. HSV-1 affects glutamate ionotropic receptors AMPA type *GRIA1* and *GRIA3*, glutamate ionotropic receptors delta type *GRID1* and *GRID2*, and glutamate ionotropic receptors kainate type *GRIK1, GRIK2, GRIK3*, and *GRIK4* (Fig. 8). *NTRK2* (Zagrebelsky et al. 2020), *KIF1A* (Fan and Lai 2022), and *PDLIM5* (Herrick et al. 2010) genes regulate dendritic spine formation, and were downregulated in all the conditions (Fig. 6).

### Effect of HSV-1 infection on cholesterol biosynthesis

**Fig. 9:**
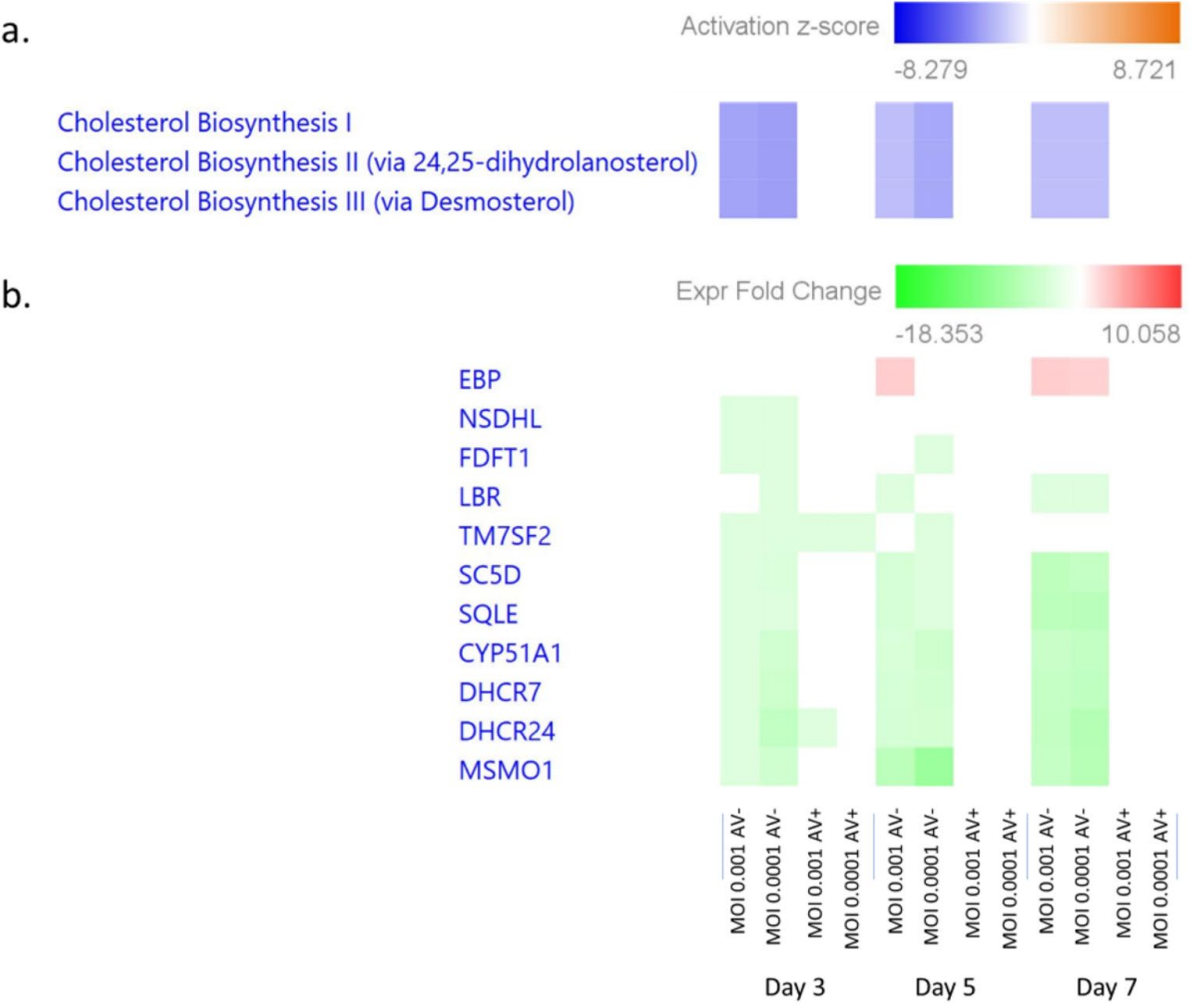
Panel **A**: Dysregulation cholesterol biosynthesis pathways after infection with HSV-1 across all conditions. Panel **B**: Dysregulation of specific genes regarding the cholesterol pathways

Consistent with previous findings, cholesterol biosynthesis was significantly downregulated in HSV-1-infected neurospheres (Blanc et al. 2011; Wang et al. 2020; Sviridov and Bukrinsky 2014; Prabhu et al. 2016; Dai et al. 2022; Chen et al. 2023; Takano et al. 2011; Ma et al. 2022; Huang et al. 2023; Wudiri and Nicola 2017; Cagno et al. 2017). In fact, on day 3 p.i. at MOI 0.001 in the absence of antivirals IPA analysis showed that the superpathway of cholesterol biosynthesis (*p* = 1.90E-0; z-score= -3.606), as well as the cholesterol biosynthesis I, II, and III pathways (*p* = 3.33E-08; z-score =-3), were most significantly downregulated. Similarly, at the same time point at MOI 0.0001, IPA analyses also indicated a significant downregulation of cholesterol biosynthesis I, II, and III pathways (*p* = 3.41E-08; z-score =-3) (Fig. 9). This downregulation of the sterol metabolic network was efficiently inhibited when infected cells are treated with 5BVdU + IFN-α. These results uncover a dependency role for the suppression of the sterol metabolic network in HSV-1-infected neural progenitor cells. Our findings indicate that HSV-1 infection leads to a significant reduction in the expression of genes involved in the sterol metabolic pathway, including those involved in cholesterol synthesis and transport. These findings provide new insights into the complex interactions between viruses and host cell metabolism in human CNS and suggests that targeting this metabolic pathway may represent a potential strategy for the development of antiviral therapies against HSV-1 infection.

Together, these findings shed light on how HSV-1 infection interferes with the complex regulation of neural progenitor cells that may cause some of the extensive neuropathogenesis in HSV encephalitis patients.

## Discussion

In this study, we sought to elucidate the impact of HSV-1 infection on the transcriptome of NPCs using a neurosphere-based model, with the primary goal of gaining deeper insights into the impact of HSV-1 infection on different aspects of neuronal differentiation. Through the identification of dysregulated pathways in HSV-1 infected neurospheres, both in the presence or absence of antivirals, we aimed to augment our understanding of how the virus influences key aspects of neurogenesis. Furthermore, we sought to evaluate the efficacy of antivirals in preventing defective neurogenesis.

Our analysis unveiled a significant reduction of viral transcripts during days 3 to 7 post-infection in the presence of antivirals. Interestingly, an increase of viral transcripts in cultures exposed to antivirals infected at both MOIs on day 5 p.i., followed by the most significant reduction on day 7 p.i. where the neurospheres had a minimal number of transcripts (Figs. 2 and 3), indicated a robust suppression of viral replication. These results are consistent with the outcomes of our prior study showing the ability of HSV-1 to undergo a form of silencing (Zheng et al. 2020b).

Among the ten most significant hierarchically clustered dysregulated pathways, oxidative phosphorylation, EIF2 signaling, apoptosis signaling pathways were identified as the most significantly upregulated pathways, as expected from the complex interplay between HSV-1 and the host. The cAMP responsive element binding (CREB) protein signaling emerged as the most significantly downregulated pathway, even in the presence of antivirals on day 7 p.i. (Fig. 5a). This finding underscores a crucial mechanism through which HSV-1 impacts essential aspects of the NPCs biology. Indeed, CREB plays a pivotal role in the regulation of neurogenesis and memory consolidation. NPCs derived from CREB-null mice exhibit severe defects in survival, cellular proliferation, and neurospheres formation, thereby emphasizing the indispensable role of CREB in NPCs neurogenesis (Dworkin et al. 2009). Antiviral treatment reduced the dysregulation of the CREB signaling during later stages of viral infection (days 5 and 7 p.i.).

In vertebrates, neural induction requires the inhibition of the bone morphogenic protein (BMP) signaling (Gaulden and Reiter 2008). Nonetheless, for an efficient neural induction, the involvement of FGF signaling is imperative (LaVaute et al. 2009; Marchal et al. 2009), and this critical pathway is downregulated in HSV-1 infected NPCs. Furthermore, FGF signaling is a critical regulator of hippocampal neurogenesis. Reduced FGF signaling leads to decreased neurogenesis (Kang and Hébert 2015). The downregulation of the EGF may cause decreased proliferation of NPCs (O’Keeffe et al. 2009) and affects their differentiation. Specifically, it promotes astroglial differentiation of NPCs (Kuhn et al. 1997).

Our analysis revealed other mechanisms by which HSV-1 can impact NPCs proliferation and differentiation, specifically involving the downregulation of *WNT*/β-catenin pathway, *STAT3, EGF*, and Sonic hedgehog signaling. The *WNT*/β-catenin pathway, a highly conserved signaling pathway that regulates key cellular functions including proliferation, differentiation, migration, genetic stability, apoptosis, and stem cell renewal (Pai et al. 2017), plays a crucial role in controlling the balance of NPC proliferation and differentiation (Gao et al. 2021). Therefore, the downregulation of the *WNT*/β-catenin pathway in HSV-1-infected NPCs may have significant consequences for the regulation of NPC proliferation and differentiation. mTOR, a downstream target of the *PI3K*/*AKT* pathway plays a pivotal role in regulating the proliferation of neural precursor cells mediated by the epidermal growth factor receptor (EGFR). Inhibition of mTOR blocks EGF-induced NPCs proliferation induced by EGF, both in vitro and in vivo (Cochard et al. 2021).

An important aspect of neurogenesis is the migration of NPCs, which is crucial for the proper lamination of the cerebral cortex, survival and differentiation of neural stem cells, expansion of neural progenitor cells, and integration into the central brain. The downregulation in infected neurospheres of genes playing an important role in the regulation of the NPCs migration, such as *ROBO1* (Gonda et al. 2013), *ROBO2* (Guerrero-Cazares et al. 2017), *SLIT1* (Deboux et al. 2020), and *ASTN1* (Wilson et al. 2010), offers valuable insights into the mechanisms by which HSV-1 affects the intricate processes involved in neural progenitor cell migration.

The expression levels of genes regulating the oligodendrogenesis, such as *MSX1, HES5, MYT1, OLIG2*, and *ID4* in HSV-1-infected neurospheres suggest that the virus may induce the differentiation of NPCs into oligodendrocytes instead of neurons. However, at day 3 p.i. and MOI 0.0001, *ID2*, known to inhibit oligodendrocytes progenitor cell differentiation, is upregulated. Additionally, at day 3 p.i. and under both MOIs, *DLX2*, a modulator of neurons versus oligodendrocytes, is upregulated. Overall, it is possible that the net effect of the opposing gene expressions may not result in the promotion of oligodendrocyte differentiation of NPCs in response to HSV-1 infection. This possibility is consistent with the finding that HSV-1 infection of NPCs did not exert any influence on the proportion of differentiating astrocytes and oligodendrocytes (Chucair-Elliott et al. 2014).

Our analysis showed that HSV-1 infection significantly downregulated elements of the cholesterol biosynthesis pathway (Fig. 9). In particular, *DHCR7* and *DHCR24* genes, which catalyze the conversion of 7-dehydroxycholesterol (7DHC) to cholesterol and the reduction of the C24 double bond in desmosterol to cholesterol (Luu et al. 2015; Prabhu et al. 2016), respectively, were robustly downregulated. Recent reports increasingly indicate that reduction of cholesterol levels by metabolic reorganization represent a host defense antiviral mechanism. Specifically, downregulation of most of genes involved in the sterol biosynthesis have been reported in primary macrophages infected with HSV-1, Semliki Forest virus (SFV), Vaccinia virus (VV), or Adenovirus (Ad) (Blanc et al. 2011). *DHCR7* inhibitors have been shown to inhibit Vesicular Virus Stomatitis (Korade et al. 2022), coronavirus (Dai et al. 2022), and Zika virus (Chen et al. 2023). The inhibition of *DHCR24* decreases hepatitis C virus (HCV) and Bovine viral diarrhea virus (BVDV) replication (Takano et al. 2011; Ma et al. 2022). The treatment of cells with methyl-β-cyclodextrin, a cholesterol-sequestering drug and the use of genetically modified cells have shown that that cholesterol is important at different stages of HSV-1 infection (Wudiri and Nicola 2017). Interferon signaling has been shown to be essential of reducing the activity of the sterol metabolic network during infection (Blanc et al. 2011). The observed downregulation of the cholesterol biosynthesis pathway in infected NPCs provides additional evidence supporting the notion that this downregulation is part of a host antiviral response (Huang et al. 2023; Wudiri and Nicola 2017; Cagno et al. 2017).

Following antiviral treatment (days 5 and 7 p.i.), the expression levels of most dysregulated genes in infected cells reverted to a relatively normal state. This indicates that the antiviral treatment effectively restored gene expression to a state closer to that of uninfected cells. However, although most of the genes returned to a relatively normal expression level due to regulatory mechanisms attempting to restore homeostasis, this may not be sufficient to completely rescue the impairment of aspects of neurogenesis.

The HSV-1 protein VHS protein, encoded by the HSV UL41 gene, causes suppression of host gene expression through an elevated global mRNA degradation rate in the cytoplasm endoribonucleolytic cleavage of target RNAs (Smiley et al. 2001; Elgadi et al. 1999). However, our analysis showed that the number of upregulated genes in cultures infected at MOI 0.001 on day 3 p.i. was comparable the number of downregulated genes (702 and 917, respectively, as shown in Fig. 4). Furthermore, the number of upregulated genes (1037) was less than halved than downregulated genes (2610), but still considerable at MOI of 0.0001 at the same time point (Fig. 4). 3.7% of the upregulated genes play a role in neurogenesis, indicating an attempt from the cells to strengthen the neurogenesis. However, it is also plausible that HSV-1 alters the expression of these genes to create a more conducive environment for its replication. In the presence of antivirals the number of upregulated genes was comparable at both MOIs.

## Conclusions

In summary, we provide an extensive analysis of transcriptomic alterations induced by HSV-1 in model NPC cultures, shedding light on the intricate mechanisms underlying the putative link between HSV-1-induced neurogenesis impairment and its effect on cognition. This analysis has unveiled significant insights into the impact of HSV-1 on critical signaling pathways involved in NPC biology. Additionally, it has shed light on the dysregulation of the cholesterol biosynthesis pathway, which has garnered increasing support as a potential host antiviral defense mechanism. We also provide evidence for the incomplete efficacy of a combined antiviral in mitigating the dysregulation of genes playing a pivotal role in the regulation of NPCs neurogenesis. Overall, this study enhances our knowledge of the complex interplay between HSV-1 and NPCs, leading us to further understanding the effects of HSV-1 on cognition.

## Materials and methods

### Virus preparation

A KOS-based recombinant virus in which enhanced green fluorescent protein (EGFP) and monomeric red fluorescent protein (RFP) are reporters whose expression is driven by the viral promoters ICP0 and Glycoprotein C, respectively (HSV-1 DualFP) (Zheng et al. 2020a) was employed in this study. The virus stock was prepared in the D’Aiuto laboratory at the University of Pittsburgh. 80–90% confluent monolayers of Vero cells were infected at a multiplicity of infection (MOI of 3 in DMEM medium supplemented with 2% FBS). After 2 h the inoculum was removed, cells were washed and cultured for 2–3 days, until the appearance of full cytopathic effect (CPE). The cells were scraped and transferred along with the culture supernatant into 15 ml conical tubes. Cells were centrifuged at 1000 rpm for 5 min. The culture supernatant was removed, leaving behind 1.5 ml, and the cell pellet was resuspended using a vortex for 1–2 min. Cells were freeze-thawed three times. Debris was then removed by centrifuging at 3000 rpm for 5 min and the top culture supernatant containing cell-free viral particles was stored at − 80 °C until use. Virus titers were determined by the standard plaque assay as described below.

### Generation of uninfected and HSV-1 infected neurosphere

Human-induced pluripotent stem cells (hiPSCs) were cultured in mTesR™ plus on Matrigel-coated tissue culture-treated plates (STEMCELL Technologies). The hiPSCs were established at the National Institute of Mental Health (NIMH) Center for Collaborative Studies of Mental Disorders-funded Rutgers University Cell and DNA Repository (RUCDR) (http://www.rucdr.org/mental-health). The control steps included the analysis of pluripotency markers NANOG, Oct4, TRA60, TRA811, OSX2 and SSEA4. We subsequently conducted karyotyping, array comparative genomic hybridization (aCGH) assays and short tandem repeat (STR) profiling and compared them with donor genomic DNA to evaluate structural changes in genomic DNA during the generation of hiPSC lines.

Human NPCs were derived from hiPSC line 73–56010-02 as previously described (Zheng et al. 2022). Briefly, hiPSCs were cultured in mTeSR1-plus medium supplemented with dual SMAD inhibitors SB 431542 and LDN 193189 to promote neural induction. After 8–10 days, neural rosettes were manually isolated, transferred into Matrigel coated plates and cultured in StemDiff Neural Progenitor Medium (STEMCELL Technologies) for the expansion of NPCs. The expression of the NPCs markers SOX1 and PAX6 was analyzed (Fig. 1). All cells were cultured in standard conditions (37 °C, 5% CO2, and 100% humidity).

Neural progenitor cells (NPCs) were seeded onto 12-well matrigel-coated plates and cultured in STEMdiff™ Neural Progenitor (NP) medium until they were 80% confluent. On the day of infection, cells were infected with HSV-1 DualFP at MOI 0.001 and 0.0001 with or without the presence of antivirals (E)-5-(2-bromovinyl)-2′-deoxyuridine (5BVdU; 30 µM) and alpha interferon (IFN-α; 125 U/ml) (*N* = 3). The media for uninfected treated control wells were switched to StemDiff™ NP medium supplemented with 5BVdU + IFNalpha at the same time. One hour later, the infectious inocula were removed and culture wells were gently rinsed once with PBS. Corresponding media were added afterwards, and cells were manually dissociated and transferred into low-attachment 6-well plates. For each well one million cells were seeded. The conditions were: (i) MOI 0.001 treated with 5BVdU + IFN-α; (ii) MOI 0.0001 treated with 5BVdU + IFN-α; (iii) uninfected but treated with 5BVdU + IFN-α; (iv) MOI 0.001 untreated; (v) MOI 0.0001 untreated; (vi) uninfected and untreated (*N* = 3). Low-attachment plates were left on an orbital shaker in the incubator to form homogenous neurospheres. In total there were three sets containing all the conditions described above, they were harvested on Day 3 post infection, Day 5 post infection and Day 7 post infection, respectively. For each replicate well of each condition, we collected all spheres (or degenerating pieces for those infected but untreated on Day 7) along with all the media from the culture well. They were centrifuged at 10,000 rpm for 2 min and the supernatants were transferred, and the pellets kept at -80˚C. Pellets were dissociated and lysed with 200µL Buffer RLT plus provided in Qiagen RNeasy plus mini kit. Samples were kept at -80˚C until further RNA extraction based on the manufacturer’s instructions.

### RNAseq

Total RNA libraries were generated using the Illumina TruSeq Stranded Total RNA Sample Preparation Guide, Revision E. The first step involved the removal of ribosomal and mitochondrial RNA using biotinylated, target-specific oligomers combined with Ribo-Zero rRNA removal beads. Following purification, remaining RNA was fragmented using divalent cations under elevated temperature, which were then copied into first strand cDNA using reverse transcriptase and random primers, followed by second strand cDNA synthesis using DNA Polymerase I and RNase H. Subsequently, a single adenosine base was added to each of the cDNA fragments, followed by ligation of an adapter. The products were purified and enriched with PCR to create the final cDNA library. A total of 12 cDNAs (two MOIs, two treatments ×three sample times) were generated. The cDNA libraries were validated using KAPA Biosystems primer premix kit with Illumina-compatible DNA primers and Qubit 2.0 fluorimeter. Quality was examined using an Agilent Bioanalyzer Tapestation 2200. The cDNA libraries were pooled at a final concentration of 1.8pM. Cluster generation and 100 bp paired-read dual-indexed sequencing was performed on Illumina NExtseq 500 (Children’s hospital of Pittsburgh, University of Pittsburgh). Sequencing read quality was assessed using fastQC v0.11.4 and CLCbio v11.0.1 software. The average number of reads per sample was 39.5 million (SD = 4.8 million reads) (Fig. 2).

Sequences were trimmed based on quality score using the modified-Mott trimming algorithm as implemented in CLC bio software, using a trim cutoff error probability of 0.05. Ambiguous bases were trimmed using a post trim maximal ambiguous base cutoff of 2. The trimmed reads were then mapped to the human genome GRCh38/hg38, using sequence and annotation provided by Ensembl (release 82). Approximately 92% of reads were mapped in pairs (SD = 1.14) across all samples, and 97.7% of reads were mapped in total (SD = 0.45).

Following human mapping, the remaining unmapped reads were collected and mapped to the Human Herpesvirus strain KOS genome (GenBank: JQ780693.1) in a similar manner.

### Bioinformatics

Functional analysis of differentially expressed genes (DEG) was performed using Qiagen’s Ingenuity Pathway Analysis (IPA, Qiagen Bioinformatics, https://www.qiagenbioinformatics.com/products/ingenuity-pathway-analysis/). IPA provides tools to interpret DEG datasets in the context of biological pathways^41^. Canonical pathway analysis identified biological pathways from the IPA library of canonical pathways that were most significant in relation to the R430-treated DEG data set. The significance of the association was measured by (1) a ratio (the number of genes from the data set mapped to the pathway divided by the total number of genes present in the pathway-map) and (2) a p-value, calculated by Fisher’s exact test. Pathways Activity Analysis, a function of IPA, enables prediction of the overall activation/inhibition states of the canonical pathways based on a z-score algorithm.

Genes involved in aspects of neurogenesis were compiled from the Gene Ontology (GO) Consortium resources (http://geneontology.org/). GO terms involved in neuronal differentiation, migration and proliferation were compiled, and these functional groupings of genes were assessed with regard to their dysregulation in HSV-1 infected cells compared to uninfected cells, both in the presence of and absence of antivirals.

## Data Availability

RNA seq data were submitted to GEO, accession number GSE236646, release date Jul 31, 2024.
